# Personalization of the Microbiota of Donor Human Milk with Mother’s Own Milk

**DOI:** 10.3389/fmicb.2017.01470

**Published:** 2017-08-03

**Authors:** Nicole T. Cacho, Natalie A. Harrison, Leslie A. Parker, Kaylie A. Padgett, Dominick J. Lemas, Guillermo E. Marcial, Nan Li, Laura E. Carr, Josef Neu, Graciela L. Lorca

**Affiliations:** ^1^Division of Neonatology, Department of Pediatrics, College of Medicine, University of Florida, Gainesville FL, United States; ^2^Department of Microbiology and Cell Science, Genetics Institute, Institute of Food and Agricultural Sciences, University of Florida, Gainesville FL, United States; ^3^College of Nursing, University of Florida, Gainesville FL, United States; ^4^Department of Health Outcomes and Policy, College of Medicine, University of Florida, Gainesville FL, United States

**Keywords:** mother’s own milk, restoration, personalization, donor breast milk, bacterial load, human milk microbiome

## Abstract

The American Academy of Pediatrics recommends that extremely preterm infants receive mother’s own milk (MOM) when available or pasteurized donor breast milk (DBM) when MOM is unavailable. The goal of this study was to determine whether DBM could be inoculated with MOM from mothers of preterm infants to restore the live microbiota (RM). Culture dependent and culture independent methods were used to analyze the fluctuations in the overall population and microbiome, respectively, of DBM, MOM, and RM samples over time. Using MOM at time = 0 (T0) as the target for the restoration process, this level was reached in the 10% (RM-10) and 30% (RM-30) mixtures after 4 h of incubation at 37°C, whereas, the larger dilutions of 1% (RM-1) and 5% (RM-5) after 8 h. The diversity indexes were similar between MOM and DBM samples, however, different genera were prevalent in each group. Interestingly, 40% of the bacterial families were able to expand in DBM after 4 h of incubation indicating that a large percentage of the bacterial load present in MOM can grow when transferred to DBM, however, no core microbiome was identified. In summary, the microbiome analyses indicated that each mother has a unique microbiota and that live microbial reestablishment of DBM may provide these microbes to individual mothers’ infants. The agreement between the results obtained from the viable bacterial counts and the microbiome analyses indicate that DBM incubated with 10–30% v/v of the MOM for 4 h is a reasonable restoration strategy.

## Introduction

The benefits of human milk for preterm infants include immune and nutritional protection against infection, decreased necrotizing enterocolitis (NEC), and other morbidities (Wight, 2001). In the absence of mother’s own milk (MOM), the American Academy of Pediatrics recommends using DBM over formula in very preterm infants (Moretti, 2012). Thus, the current practice at the University of Florida, UF Health Neonatal Intensive Care Unit (NICU), is to provide premature infants of gestational age less than 30 weeks with DBM if the MOM supply is low or not available.

Numerous components of human milk are thought to be beneficial for the infant (Newburg et al., 2005). Accumulating data suggests that microbes that are indigenous to human milk may not be contaminants and play a beneficial role for the infant (Jeurink et al., 2013). Up to 200 different bacterial species have been found in human milk. Hunt et al. (2011) studied milk samples from 16 healthy women collected at three different time points. A common group of nine bacterial genera was present in all samples, but in different concentrations among the subjects. Each individual demonstrated a unique milk microbiome that was stable over time (Hunt et al., 2011). Collectively, these data highlight a personalized collection of milk microbes from each mother that is optimized for the health of her own infant.

Donor breast milk used in most NICUs is pasteurized due to safety concerns (Landers and Updegrove, 2010). Pasteurization of DBM kills 99% of bacteria and may also inactivate a large proportion of the bioactive components. Since DBM is pooled and pasteurized, it lacks the unique live maternal milk microbiome, which may be of benefit to the infant (for a review see Jost et al., 2015). The majority of mothers of very preterm infants are able to express small amounts of their own milk and although it may be of insufficient volume to meet the daily nutritrional requirements of their infant, it can still provide lasting health benefits. Our objective was to encourage the NICU mothers to continue pumping and use a small amount of MOM to inoculate the pasteurized DBM to add back the potentially beneficial naturally occurring microbes. We hypothesized that fresh MOM can be mixed with DBM to improve the quality of DBM by cultivating the beneficial milk microbiome to reflect MOM. Studies were performed to determine optimal dilutions and incubation times to obtain a microbial content most similar to that of the MOM.

## Materials and Methods

### Subjects

The study cohort consisted of twelve mothers who provided a breast milk sample between December 2014 and February 2016. Mothers had delivered an infant at less than 32 weeks gestation, weighing less than 1500 g at birth, and who were expressing over 100 mL of breast milk per day and producing at least 45 mL with each expression session. This pilot study was approved by the Institutional Review Board (IRB201400527) at the University of Florida and mothers provided written informed consent. Exclusion criteria included mothers who had delivered an infant with a chromosomal abnormality or who was severely ill and mothers who were currently taking antibiotics. Data collected included gender, gestational age at birth, gestational age at breast milk collection, birth weight, race, maternal age, parity, mode of delivery, maternal BMI, maternal infections, maternal medications, maternal antibiotics, and Medicaid eligibility. Baseline demographics are summarized in **Table 1**.

**Table 1:** Demographics of mothers and infants.

Infant demographics (*n* = 12)	
Gestational age at birth (weeks)	27 ± 2.67
Birth weight (grams)	951.83 ± 397.02
Post-menstrual age at sample collection (weeks)	31 ± 2.86
Gender	
Male	75% (*n* = 9)
Female	25% (*n* = 3)

**Maternal demographics**	

Maternal age (years)	27 ± 4.95
Medicaid eligible	50% (*n* = 6)
Delivery	
C-section	42% (*n* = 5)
Vaginal	58% (*n* = 7)
Maternal BMI	28.35 ± 5.44
Maternal antibiotics	92% (*n* = 11)
Breastfeeding or attempts prior to milk sample	25% (*n* = 3)

**Mother/Infant**	

Race	
Caucasian	58% (*n* = 7)
African American	25% (*n* = 3)
Hispanic	17% (*n* = 2)

### Milk Collection

Each mother pumped one sample of 45 mL of breast milk (MOM) into a sterile container using a new breast pump kit (Symphony Breast Pump Kit, Medela LLC, McHenry, IL, United States) and hospital grade electric breast pump (Symphony Breast Pump, Medela LLC, McHenry, IL, United States). Prior to sample collection, mothers were provided the verbal instructions regarding hand hygiene during milk expression and techniques for breast pump cleaning per NICU protocol. Immediately following collection, the samples were placed on ice and delivered to the laboratory for processing. The pasteurized DBM was obtained frozen from the Human Milk Banking Association of North America (HMBANA) milk bank (see http://rmchildren.org/mothers-milk-bank/donate-milk/collection-and-storage/ for details on the collection guidelines for donors) and were thawed immediatly prior to each restoration process.

### Restoration of the Live Microbiome of DBM

The restoration strategy was to add increasing amounts of MOM (1, 5, 10, and 30% v/v) into pasteurized DBM. As controls, pure DBM and MOM were included. Once blended, the milk mixtures were incubated at 37°C. Samples were taken at time 0, 4, and 8 h. For viable bacterial counts, samples were analyzed immediately. For microbiome analyses, 2 mL were centrifuged at 12,000 rpm for 5 min at 4°C, the supernatant removed, and pellets stored at -80°C until the DNA was extracted. The pH and physical appearance of the samples at each time point was also recorded.

### Culture Dependent Bacterial Analysis

Viable cell counts were determined by plating serial dilutions of each sample on selective and non-selective agar plates at time 0, 4, and 8 h (T0, T4, and T8, respectively). Based on the most common groups of bacteria cultivated from human milk (Wight, 2001; Martín et al., 2003; Reviriego et al., 2005; Jost et al., 2013; Menon and Williams, 2013), the following media were used: acidified Man, Rogosa and Sharp (MRS) agar for lactic acid bacteria (Fisher Scientific Company), Berens agar (BSM agar) for *Bifidobacterium* (Sigma–Aldrich) amended with BSM supplement Mupirocin Lithium Antibiotic (Fisher Scientific Company), Mannitol salt agar (MSA) for *Staphylococcus* (Fisher Scientific Company), Nutrient rich agar for facultative aerobes including *Streptococcus* (Sigma–Aldrich), and MacConkey agar for enterobacteria (Fisher Scientific Company). All plates were incubated at 37°C for 48–72 h. MRS and BSM agar plates were incubated in jars enclosing a burning candle to create a reduced oxygen environment while MacConkey and Nutrient agar plates remained incubated aerobically.

### DNA Isolation, Library Construction and Sequencing

DNA was extracted from milk samples and preserved at -80°C using the PowerFecal^®^ DNA isolation kit (MoBio Lab, Inc. United States) with the following modification: the pellet was homogenized in 750 μL of bead solution, then 100 μL of Protease from *Streptomyces griseus* 20 mg/mL (Sigma–Aldrich, Steinheim, Germany) was added (Marcial et al., 2017) The mixture was incubated at 37°C for 15 min, then the samples were processed according to the manufacturer’s protocol. In the elution step, the DNA was collected in 70 μL of water and quantified. The DNA concentration was standardized to 1 ng/μL before the amplification of the V4 region using primers 515F/806R barcoded for Illumina HiSeq platform (Caporaso et al., 2012). To reduce variability and potential bias from potential sources of DNA contamination, all samples were processed with the same batch of DNA extraction kits as well as PCR reagents.

### Bioinformatics and Statistical Analysis

Clustering of Operational Taxonomic Units (OTUs) at 97% similarity was performed with the subsampled open-reference OTU picking method (Rideout et al., 2014) with no removal of singletons. The Greengenes reference dataset version 13.8 (DeSantis et al., 2006) was used as the reference for OTU picking and for taxonomy assignment with uclust (Edgar, 2010). OTUs identified as mitochondrial DNA or as chloroplasts were removed from further analyses using R studio.

Community structure was analyzed in R with phyloseq (McMurdie and Holmes, 2013) and plotted with ggplot2 (Wickham, 2009). Differences in taxonomic profiles were analyzed by Welch’s *t*-test (for two groups) or by ANOVA (for multiple groups) with Tukey-Kramer *post hoc* tests with STAMP (Parks et al., 2014) and PAST (Hammer et al., 2001).

## Results

### Mother’s Own Milk Shows a High Variability in the Number of Culturable Bacteria

**FIGURE 1:**
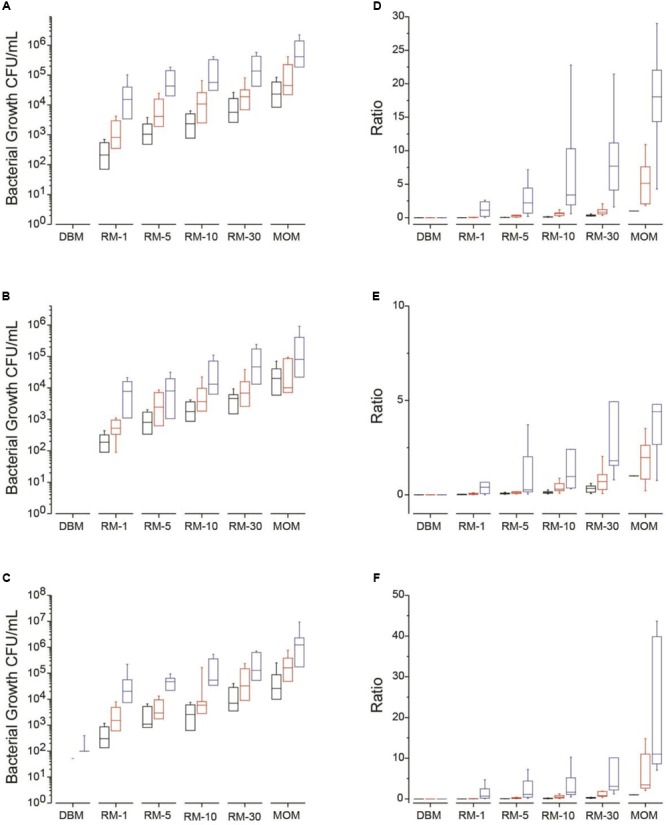
Median bacterial growth (CFU/mL) for each milk sample (DBM, RM-1, RM-5, RM-10, RM-30, and MOM) on different media types at time = 0 (T0, black boxes) and incubated over 4 h (T4, red boxes) and 8 h (T8, blue boxes) at 37 °C. Bacterial growth was determined on **(A)** MSA, **(B)** MRS and **(C)** on Nutrient media. Mean ratios of bacterial growth of RM samples to MOM **(D–F)**. The ratio was calculated for each RM sample by dividing the CFU/mL in each RM sample to its corresponding MOM sample at T0 (MOM = 1). The ratios were calculated at each time point in **(D)** MSA, **(E)** MRS, and **(F)** Nutrient media.

At baseline *t* = 0, the amount of four bacterial populations were quantified in MOM and used as the target goal for each individual mother. It was found that the amount of bacteria in MRS plates (lactic acid bacteria including *Lactobacillus*) were between 10^3^ and 10^5^ CFU/mL with most of them being at 10^4^ CFU/mL (**Figure 1B**). The bacterial load in MSA and nutrient broth were between 10^3^ and 10^6^ CFU/mL with an equal distribution in concentrations among the MOMs (**Figures 1A,C**). Only four MOM samples (30% of samples) grew on MacConkey agar at concentrations between 10^1^ and 10^2^ CFU/mL (data not shown). *Bifidobacterium* colonies were not recovered on Berens agar under our experimental conditions. The viable counts were also determined in DBM. Around 10^2^ CFU/mL were counted in nutrient broth for half of the DBM samples (**Figure 1C**).

### Restoration of the Live Microbiome of DBM with Mother’s Own Milk

As described in the methods section, each of the twelve samples of MOM were inoculated in DBM at 1% (RM-1), 5% (RM-5), 10% (RM-10) and 30% (RM-30). Samples, including incubated DBM and MOM, were taken across three time points. The goal of this experiment was to determine the cultivable bacterial load of each sample to establish the minimum time and dilution required to reach the initial bacterial concentration found in MOM.

For each growth media, the concentration of bacteria was determined and ratios were calculated using MOM concentration at time 0 as the target concentration (represented as 1) (**Figures 1D–F**). On average, all RM samples increased in bacterial concentration over time (**Figures 1A–C**). A good correlation was found between the size of the inoculum and the amount of bacterial growth while the initial concentration of bacteria did not affect the outcome.

For MSA media, which targets mostly *Staphylococcus*, after 4 h of incubation at 37°C, 75% of the RM-10 reached a ratio of 0.6 compared to the MOM original bacterial load while all RM-30 reached a ratio of 1. For MRS media, which targets mostly lactic acid bacteria, 33% of the RM-10 reached a ratio of 1 compared to MOM while 58% of RM-30 reached the same ratio after 4 h. In Nutrient agar, which is a general purpose media targeting non-fastidious organisms, after 4 h of incubation 42% of samples in RM-10 reached a ratio of 1 compared to MOM while 83% reached a ratio of 1 in the RM-30 samples. After 8 h of incubation the bacterial load in all growth media tested (MSA, MRS, and Nutrient agar) for RM-10 and RM-30 went over the initial concentration of MOM (**Figure 1**). In contrast, the two highest milk dilution ratios (RM-1 and RM-5) were less than 0.5 at 4 h of incubation and then at 8 h of incubation reached a ratio > 1, exceeding the MOM bacterial load (**Figure 1**).

Since microbial growth may result in changes in pH as well as physical changes to the milk, the overall appearance (i.e., phase separation and curdling) and pH was monitored throughout the incubation period. Visual inspection of the milk samples did not reveal changes during the incubation period. Analysis of pH indicated that MOM samples were more alkaline (pH 7.5 ± 0.11) than pasteurized DBM (pH 6.5 ± 0.15) at T0. The incubation of MOM milk for 8 h resulted in a significant decrease (*p* = 0.047) in pH to 7 ± 0.5 while no significant changes were observed in DBM samples or RM samples over time.

### Donor Milk versus Mother’s Own Milk Have a Similar Diversity Index

Illumina sequencing of the V4 region of the bacterial 16S rRNA was performed on all milk samples. After quality control, a total of 15,575,142 sequences were obtained with a mean of 75,976 sequences per sample. The rationale of this microbiome analyses was to have an unbiased view of the changes in the microbial community during the restoration process.

**FIGURE 2:**
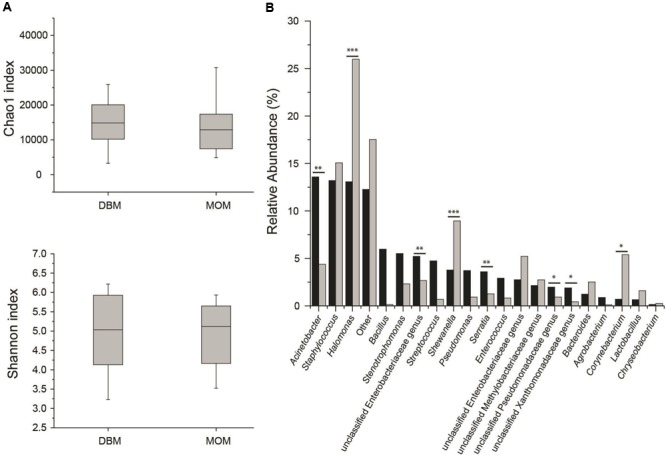
**(A)** Alpha diversity determinations of DBM and MOM at T0 using Chao1 and Shannon indexes. **(B)** Summary of the most abundant genera found in DBM (black) and MOM (gray) samples. Statistically significant values are indicated ^∗∗^*p* < 0.05 and ^∗∗∗^*p* < 0.01. Differences at ^∗^*p* < 0.1 are also indicated.

First, we compared the community structure of DBM and MOM at T0. The alpha diversity expressed as Chao1 and Shannon index was similar between the two sets of samples (**Figure 2A**). It cannot be concluded, however, that due to the similarity in alpha diversity between DBM and MOM that the bacterial load is the same since DBM is pasteurized. The relative abundance of genera was compared between the two samples (**Figure 2B**). *Acinetobacter, Staphylococcus, Halomonas, Bacillus, Stenotrophomonas, unclassified Enterobacteriaceae genus, Streptococcus, Shewanella, Pseudomonas, Serratia, Enterococcus*, unclassified Enterobacteriaceae genus, unclassified Methylobacteriaceae genus, unclassified Pseudomonadaceae genus, unclassified Xanthomonadaceae genus and *Bacteroides* constituted 85% of the sequences found in DBM. In MOM the most abundant genus were *Halomonas, Staphylococcus, Shewanella, Corynebacterium*, Enterobacteriaceae genus, *Acinetobacter*, unclassified Methylobacteriaceae genus, unclassified Enterobacteriaceae genus, *Bacteroides, Stenotrophomonas* and *Lactobacillus*. The statistical analyses showed that *Halomonas* (*p* < 0.01) and *Shewanella* (*p* < 0.01) were more abundant in MOM than in DBM samples. Similarly, *Staphylococcus, Corynebacterium*, and *Lactobacillus* were more abundant in MOM, yet did not reach statistical significance (*p* < 0.1) when compared to that of DBM. On the contrary, *Acinetobacter*, an unclassified Enterobacteriaceae genus, and *Serratia*, showed a significantly higher relative abundance (*p* < 0.05) in DBM.

### Fluctuations in the Alpha Diversity of RM Samples during Microbial Restoration

**FIGURE 3:**
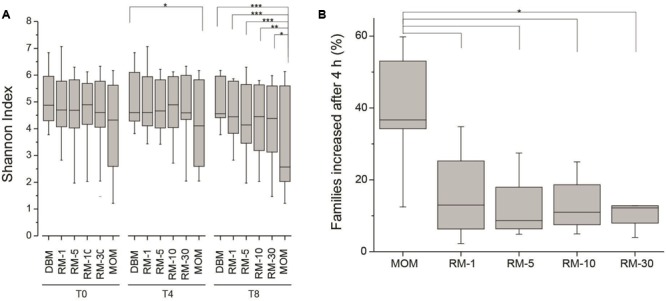
**(A)** Alpha diversity assessment in RM samples (RM-1, RM-5, RM-10 and RM-30, across time points (0, 4, and 8 h). DBM and MOM samples were included in the analyses. It was found that DBM and MOM are significantly different from each other at T4 and T8. The alpha diversity of the 30% dilution of MOM at T4 resembles that of MOM at T0. Statistically significant values are indicated as ^∗^*p* < 0.05, ^∗∗^*p* < 0.01, and ^∗∗∗^*p* < 0.001. **(B)** Summary of the expansion of bacterial families after 4 h of incubation. The expansion of bacterial families was determined for each sample by substracting the relative abundancy at T4 from the relative abundancy at T0. Statistically significant values are indicated as ^∗^*p* < 0.001.

Next, the fluctuations in the microbial community as a result of the restoration process was determined. As described earlier, the working hypothesis is that the restoration process will result in the expansion of the microbial population without loss of diversity. The expansion of MOM was used as a positive control to determine microbial populations that will be able to expand *in vitro*. The Shannon index was utilized to determine the species richness across time between DBM, MOM, and the RM samples (**Figure 3A**). It was found that after 4 h of incubation the MOM samples had a significant decrease (*p* < 0.01) in alpha diversity only when compared to DBM and RM-1. In contrast, after 8 h of incubation, there is a significant decrease (*p* < 0.001) in diversity when comparing MOM to all other samples (DBM and RM). However, even when a decrease in diversity was observed in RM-10 and RM-30, they did not reach statistical significance (*p* = 0.07 and *p* = 0.14, respectively). The change in diversity of MOM and RM-10 or RM-30 suggests the differential growth of few bacterial species.

**FIGURE 4:**
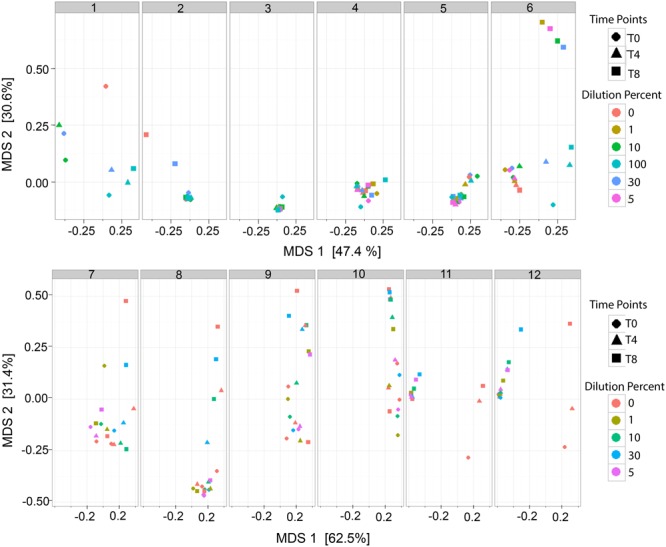
Multidimensional scaling (MDS) plots of RM samples. Two dimensional clustering determined by the distance of each sample to show individual clustering in each MOM derived subset. Each time point (T0, T4, and T8) is signified by a shape (Circle, Triangle, and Square, respectively). Each dilution is signified as a color. For MOM 1 and MOM2 samples RM-1 and RM-5 were not available. For MOM1, only DBM at T0 (DBM = 0 and T0 = circle) is shown.

The analysis of the bacterial richness of the RM allowed for the assessment of the expanding microbiome to determine if the RM samples, over time, become similar to MOM at T0. A multidimensional scaling (MDS) plot was generated to visualize the variability of the microbial community in each MOM and their derived RM samples (**Figure 4**). It was expected that the DBM samples of each individual set should remain clustered together since DBM is pasteurized and should have negative to little bacterial growth. In most cases, the DBM samples remained clustered together, such as MOM sample 4, 5, 6, 7, 8, 10, 11, and 12 (shown as circles in **Figure 4**) whereas, in DBM used for MOM samples 2 and 9 has a small difference in bacterial richness over time. Overall, these MDS plots allowed the visualization of fluctuations of the microbial population in the different RM samples over time. In some mothers, it was observed that the different RM samples migrated toward MOM at T0, suggesting that the RM sample became more similar to MOM at T0 (**Figure 4**). However, some exceptions were observed. For example, all samples derived from MOM 3 cluster together, indicative that the restoration process was not successful. Mothers 11 and 12 show migration of all the dilutions moving away from MOM at all time-points and toward DBM instead. Mothers 11 and 12 shared the same sample of DBM so it is possible that live bacteria still found within this DBM sample are hindering the expansion of MOM-derived microbiota.

### RM Samples Do Not Share a Common Expanding Microbiome

Next, we determined if a core microbiome was expanding in all the RM samples. Based on the culture dependent results where the target bacterial load (of the culturable population) was reached after 4 h and a decrease in diversity was observed in the microbiome after 8 h of incubation, the next series of analyses were performed on all the samples (DBM, RM, and MOM) only after 4 h of incubation.

For these analyses the relative abundance of each bacterial family was obtained. To identify families that increase in abundance after 4 h, the bacterial load from T0 was subtracted from the bacterial load from T4 across all samples. If a family increased in concentration within the negative control (DBM), it was excluded in the results. It was found that out of the 120 ± 40 families identified within MOM samples, an average of 37 ± 12 were able to increase concentration after 4 h of incubation. Interestingly, in all RM samples, 23–38% of the families were able to increase in concentration (**Figure 3B**).

All bacterial genera within the families that increased in concentration after 4 h of incubation were analyzed. Some genera showed some statistical trends like *Gemella*, unclassified Gemellales genus, *Salinicoccus, Gallionella*, unclassified Proteobacteria genus 1, unclassified Proteobacteria genus 2, *Shewanella, Alicyclobacillus, Pediococcus*, and *Lactobacillus*, however, no significant differences (*p* < 0.2) were observed (data not shown). In contrast, *Staphylococcus* showed a significant increase in concentrations (*p* < 0.01) (data not shown). In summary, a common core of microbes that expand in RM samples was not identified, with the exception of *Staphylococcus*.

### Mother’s Own Milk Samples Cluster by the Birth Mode of Delivery

Unable to determine a common expanding microbiome among RM samples, the data was stratified and analyzed considering the birth mode of delivery. The principal component analysis using MOM samples at T0, clustered into c-section and vaginal delivery with the exceptions of mothers 6 and 7 (**Supplementary Figure S1**). The distribution of the 15 most abundant genera among samples is shown in **Supplementary Figure S2**.

The microbiome of MOM samples divided by mode of delivery (c-section *n* = 5 and vaginal *n* = 7) were analyzed using a Welch’s *t*-test (Supplementary Table S1). It was found that *Erwinia* (*p* = 0.03) and *Pseudomonas* (*p* = 0.03) were more prevalent among c-section samples while *Halomonas* (*p* = 0.02), *Lactobacillus* (*p* = 0.04), *Prevotella* (*p* = 0.04), *Ruminococcus* (*p* = 0.04), unclassified Clostridiaceae genus (*p* = 0.03), and unclassified Enterococcaceae genus (*p* = 0.02) were found at higher concentrations in vaginal delivery samples (Supplementary Table S1). Other genera that had *p*-values greater than 0.05 and less than 0.13 were also taken into consideration for further analyses. These results indicate that the differences in genera observed between the mode of delivery of MOM might explain the inability to identify a core microbiome that expand in RM samples.

The data from RM samples was stratified by mode of delivery and the genera that were differentially found between c-section and vaginal delivery were analyzed (Supplementary Table S1). The relative increase in abundace after 4 h of incubation was tested using ANOVA and Tukey’s *post hoc* tests. It was found that most genera did not change significantly. *Agrobacter* showed a significant difference (*p* < 0.05) between vaginal RM-10 and c-section RM-10. Interestingly, 3 unclassified genera (unclassified Clostridiaceae genus, unclassified Enterococcaceae genus 2, and unclassified Methylophilaceae genus) increased in relative abundace in all c-section RM samples but not in vaginal delivery birth RM samples (data not shown). These results suggest that each MOM has a unique diverse bacterial load, having no core microbes.

## Discussion

In this work we show that by using a small amount of MOM to inoculate pasteurized DBM, it is possible to reestablish the potentially beneficial naturally occurring microbes. MOM contains irreplaceable immune modulating factors including commensal bacteria (Wold and Adlerberth, 2002; Jost et al., 2013). Feeding preterm infants MOM has been shown to decrease NEC and sepsis with even small amounts of MOM providing some protection (Furman et al., 2003; Schanler, 2005; Meinzen-Derr et al., 2009; Corpeleijn et al., 2012, 2016; Abrams et al., 2014; Chowning et al., 2016). The microbiome found in MOM may provide short term and long term benefits to infants including preventing colonization by pathogens, stimulating production of cross-reactive antibiodies, and possibly establishing a healthy intestinal microbiome which may prevent long term morbidities including obesity, type 2 diabetes, chronic intestinal inflammation, autoimmune disorders, allergy, irritable bowel syndrome, and allergic gastroenteritis (Goulet, 2015; Lemas et al., 2016; Wallace et al., 2016). Unfortunately, many mothers of preterm infants are unable to produce sufficient amounts of breast milk to sustain 100% of their infant’s nutritional needs (Smith et al., 2003; Lee and Gould, 2009). The RM may offer a personalized and beneficial alternative to DBM when MOM is limited. Since MOM contains a unique and unchanging microbiome (Hunt et al., 2011; Cacho and Neu, 2014), providing infants their own mother’s milk may be beneficial, especially for infants born preterm, at risk for infection and other premature specific morbidities.

Our culture dependent approach indicated that the main bacterial groups that were tested, namely *Staphylococcus*, lactic acid bacteria (and other that can grow in MRS agar), along with other facultative aerobes (including *Streptococcus*), can be propagated into DBM, however, a large variability in bacterial load was observed between mothers. Previous studies examining bacteria present in breast milk have required aseptic sample collection of milk by mothers to limit skin flora and potential contaminants (Martín et al., 2003, 2007; Collado et al., 2009; Hunt et al., 2011; Jost et al., 2013; Khodayar-Pardo et al., 2014). In contrast, we aimed to examine the typical microbes that the preterm babies consistently received from their mother’s milk during routine NICU expression practices and therefore, mothers performed routine NICU protocols for hand hygiene techniques and equipment cleaning. Consistent with Heikkilä and Saris (2003) and Martín et al. (2003), our results reveal that *Staphylococcus*, facultative aerobes, lactic acid bacteria, and few Gram negatives can be cultured from breast milk. We were not able to isolate *Bifidobacterium* in culture which may be due to the sensitive anaerobic nature of *Bifidobacterium*, having not provided the proper anaerobic environment or handling techniques to facilitate growth. In addition, it is possible that *Bifidobaceterium* is negligible in breast milk from mothers delivering preterm infants as indicated from the study by Khodayar-Pardo et al. (2014), which showed less *Bifidobacterium* compared to milk from mothers of term infants. Small numbers of Gram negative bacteria grew on MacConkey agar (results not shown), which is consistent with previous research indicating that approximately 30–70% of breast milk samples contained Gram negative bacteria (Botsford et al., 1986; Landers and Updegrove, 2010; Keim et al., 2013). Small amounts of Gram negative bacteria in breast milk may be important in preparing the immune system to utilize toll-like receptor mediated tolerizing mechanisms to prevent an exaggerated response to future Gram negative bacteria (Madara, 2004).

Interestingly, pasteurized DBM obtained from HMBANA showed that 10^2^ CFU/mL of bacteria were still present in nutrient broth (facultative anaerobes including *Staphylococcus* and *Streptococcus*) in 44% of the DBM samples. This is consistent with previous studies describing the presence of staphylococcal species in DBM as well as spore forming bacteria such as *Bacillus cereus* (Crielly et al., 1994; Landers and Updegrove, 2010; Decousser et al., 2013; Akindolire et al., 2015; Dewitte et al., 2015). Although microbial diversity indexes were similar between DBM and MOM, the most abundant genera differed. The most prevalent genera in MOM were *Halomonas, Shewanella, Corynebacterium, Staphylococcus*, and *Lactobacillus*, while the most common genera in DBM were *Acinetobacter*, unclassified Enterobacteriaceae, and *Serratia*. These differences may reflect variances in the mothers who provided breast milk samples and the sanitation or method used for collection. DBM is typically obtained from mothers who are breastfeeding term infants who are often more than 6 months old. In contrast, the MOM in this study was obtained from mothers of preterm, hospitalized infants who because of their infant’s prematurity, were unable to breastfeed and were thus dependent on mechanical breast milk expression to obtain milk for their infants.

We found that *Halomonas* was present in greater abundance than *Staphylococcus* in comparison with other studies in MOM. Although *Halomonas* has not been previously described in breast milk from mothers delivering preterm, *Staphylococcus* is a well-known predominant phyla in breast milk (Hunt et al., 2011; Urbaniak et al., 2012, 2016; Jost et al., 2013). We also found *Shewanellaceae* was high in MOM (*p* < 0.05) compared to DBM. The main genera found to be of great abundance in MOM across different studies are *Staphylococcus, Streptococcus*, Proteobacteria groups, and *Propionibacteria* (Hunt et al., 2011; Kumar et al., 2016). However, *Bifidobacterium, Bacteroides, Parabacteroides* and Clostridia groups have also been identified to be a part of the breast milk microbiota (Sinkiewicz and Ljunggren, 2008; Kumar et al., 2016). Although *Bifidobacterium* was identified from our sequencing results, it was not shown to be prevalent. These differences in the predominant genera being *Halomonas* and *Shewanellaceae* as opposed to *Staphylococcus* and *Streptococcus* may be due to the fact that (except for rare occasions in three patients) mothers in our study did not breastfeed their infants. They were dependent on mechanical breast pumps for milk removal, thus the mother’s breasts were not routinely exposed to oral microbes from the infant’s mouth. Other reasons for these variations may be attributed to younger gestational age at delivery, stage of lactation, milk collection strategies and geographic variations in our study compared to others. In DBM, the most abundant genera were *Acinetobacter*, unclassified Enterobacteriaceae genus, and *Serratia.*

Results of this study suggest that the optimal restoration strategy to reach a microbial content most similar to MOM was a mixture of RM-10 incubated for 4 h. Using the bacterial load and microbial content of MOM at T0 as the target, we were able to successfully restore the microbiome in the RM-10 and RM-30 mixtures after 4 h of incubation, whereas larger dilutions of RM-1 and RM-5 reached the target level after 8 h. Although it did not reach the microbial content of MOM, RM-10 was able to reach 60% of the bacterial load of MOM. In contrast, RM-30 exceeded the target goal of MOM in the majority of incubated samples. This may be clinically undesireable since potentially pathogenic bacterial strains may grow to possibly harmful levels. Overall, our results demonstrate that inoculation with an amount of MOM as small as 1% can populate DBM with the mother’s potentially beneficial bacteria.

The alpha diversity of MOM and the larger dilutions of RM (RM-10 and RM-30) decreased as incubation time increased. In contrast, the diversity of DBM and smaller dilutions of RM (RM-1 and RM-5) remained similar to their original levels as incubation time increased. This trend of MOM toward decreased diversity suggests replication of only a few microbial species. Breast milk studies show that microbial diversity is associated with a healthy lactating milk microbiome as opposed to the milk microbiome of a mother with mastitis where *Staphylococcus* or *Streptococcus* species predominate (Delgado et al., 2008). This decreased diversity indicates that in order to preserve microbial diversity similar to that found in MOM, a 4 h incubation time compared to an 8 h incubation time may be optimal, which confirms the culture based results favoring a 4 h incubation time over 8 h for the RM.

Birth mode of delivery has been shown to affect the microbiota of breast milk in the majority of studies, suggesting a difference in the milk microbiome between infants born via c-section and those born vaginally (Azad et al., 2013; Gregory et al., 2015; Liu et al., 2015; Brumbaugh et al., 2016; Dominguez-Bello et al., 2016; Lee et al., 2016; Nagpal et al., 2016; Rutayisire et al., 2016). In our study, we observed a differential clustering of microbiomes from the breast milk of c-sections versus vaginally delivering mothers. Although it is clear each individual mother’s milk microbiota has bacterial variability, c-section and vaginal deliveries cluster with one another. Further analysis illustrated that bacterial genera most prevalent in breast milk from vaginal deliveries were *Halomonas, Lactobacillus, Prevotella*, unclassified Clostridiaceae genus, *Clostridium, Comamonas*, and *Dorea.* Those genera most prevalent in breast milk from cesarean deliveries were *Erwinia, Pseudomonas, Ruminococcus*, unclassified Enterococcaceae genus, *Agrobacterium, Citrobacter, Enterococcus, Klebsiella*, unclassified Bacilli genus, unclassified Bradyrhizobiaceae genus, and unclassified Methylophilaceae genus. Further statistical analysis does not show a common microbiota across breast milk regardless of c-section or vaginal delivery, concluding that the microbiota of breast milk is variable between each mother.

Based on our results, restoration of the live microbiome of DBM with MOM appears to be a promising and innovative method to provide preterm infants with beneficial breast milk bacteria. It is well known that breast milk changes over time to meet specific needs of infants which are attributed to stages of lactation, gestational age, infant feeding, and the health status of the breastfeeding dyad (Daly et al., 1996; Kent, 2006; Hassiotou et al., 2013). If mothers of preterm infants are able to express even minimal amounts of breast milk, restoration of the microbiome in DBM may allow their infants to receive milk more specific to their individual needs based on the stage of lactation and gestational age, thereby potentially improving their overall health. We used non-culture based techniques to take a snapshot of the full range of bacteria present in fresh preterm milk and pasteurized donor milk. The main limitation of our study is its small sample size and the use of antibiotics during the peripartum period. Nevertheless, it addresses the concept that the live microbiota donor human milk can be effectively restablished by MOM. In addition, mothers were not required to clean their breasts prior to breast milk sampling so their samples may have contained a higher level of skin colonizing microbes. Another limitation of the study is that safety parameters were not assessed. Future studies will need to include the analyses of potential pathogenic bacterial groups that may proliferate in the RM samples in the NICU environment.

In summary, we have shown that each mother has a unique milk microbiota and that the live microbiome in DBM can be restored with these unique bacteria using small amounts of MOM. This is a novel approach to possibly improving the bioactivity of DBM by adding specific MOM microbes in small quantities to personalize her own infant’s milk. Personalizing DBM may benefit the mother–infant dyad and contribute to a more robust infant intestinal microbiome. The agreement between the results obtained from the viable bacterial counts and the microbiome analyses indicate that DBM incubated with 10 percent of the MOM for 4 h is a reasonable restoration strategy. Future studies should include larger samples sizes, activity of the microbes in RM in comparison to DBM and MOM samples, and clinical evaluation of the safety and efficacy.

## Ethics Statement

This pilot study was carried out in accordance with the recommendations of the Institutional Review Board (# 201400527) at the University of Florida with written informed consent from all subjects. All subjects gave written informed consent in accordance with the Declaration of Helsinki. The protocol was approved by the Institutional Review Board at University of Florida.

## Author Contributions

The authors’ responsibilities were as follows: NC, LP, JN, and GL designed the research; NC, NH, KP, GM, LC, and NL performed the research; DL contributed new reagents/analytic tools; NC, NH, LC, LP, and GL analyzed the data; NC, NH, LP, JN, and GL evaluated the data; NC, NH, LP, JN, and GL wrote the manuscript; NC, NH, LP, JN, and GL had primary responsibility for the final content; All authors read and approved the final manuscript. Medela, AG was not involved in the implementation, data collection, statistical analysis, interpretation of date, or manuscript preparation and writing.

## Conflict of Interest Statement

The authors declare that the research was conducted in the absence of any commercial or financial relationships that could be construed as a potential conflict of interest.

## Supplementary Material

FIGURE S1 This is a principal component analysis (PCA) of the microbiota of MOM samples at T0. The circles represent moms that delivered through C-Section and the squares are those that delivered through vaginal births.

FIGURE S2 Heatmap showing the top 15 genera of each DBM sample, with the exception of replicates DBM 1, 2 and 11 (DBM 1 and 2 being a replicates of 3 and DBM 11 being a replicate of 12), and each MOM sample.

## Abbreviations

DBM: donor breast milk
MOM: mother’s own milk
NICU: Neonatal Intensive Care Unit
RM: restored microbiota pasteurized donor milk.
