# Supplementary figures and images for: Personalization of the Microbiota of Donor Human Milk with Mother’s Own Milk
Source: Front Microbiol. 2017 Aug 3;8:1470. doi: 10.3389/fmicb.2017.01470 (PMC5541031; doi:10.3389/fmicb.2017.01470)

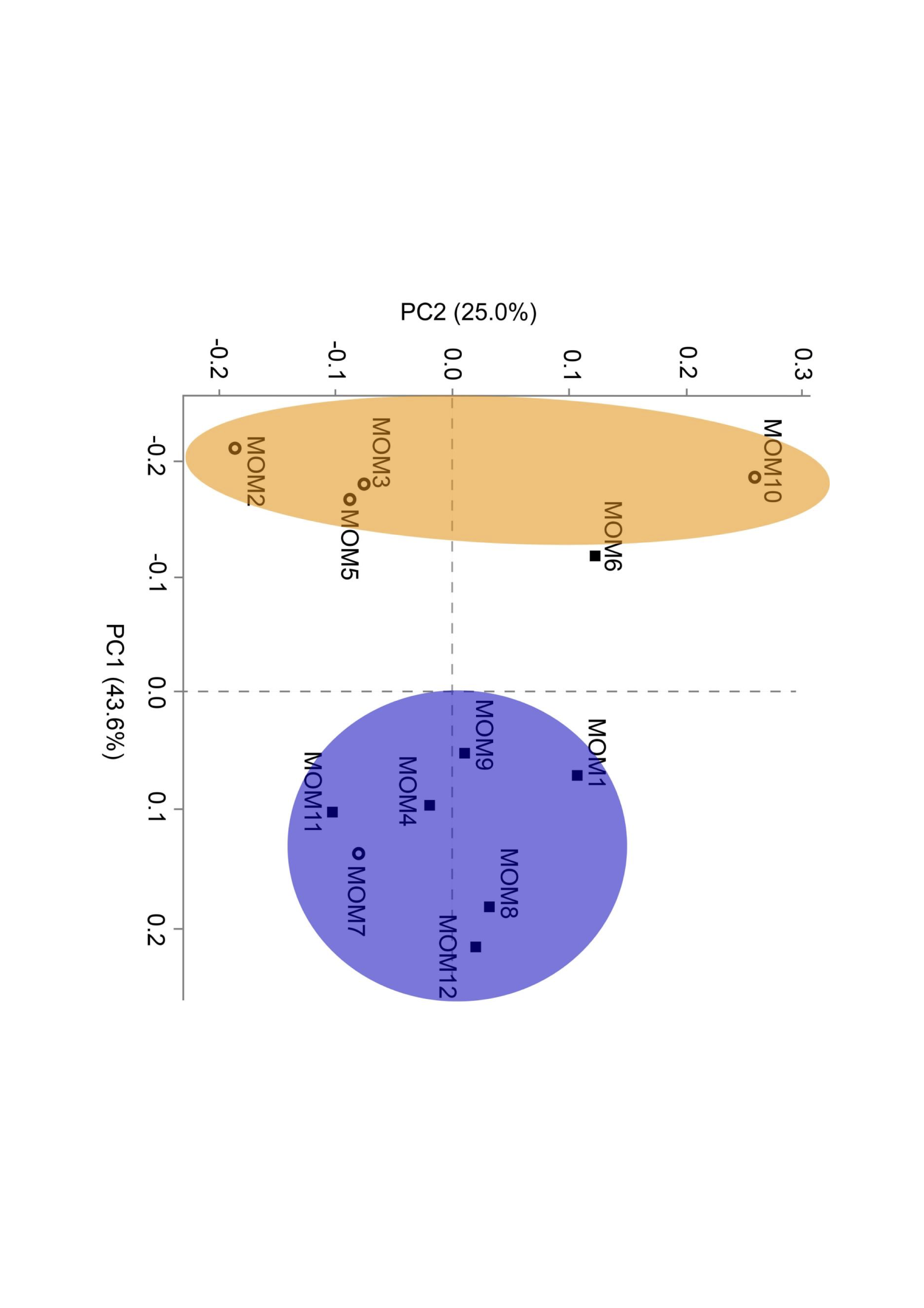

Supplement: FIGURE S1 — This is a principal component analysis (PCA) of the microbiota of MOM samples at T0. The circles represent moms that delivered through C-Section and the squares are those that delivered through vaginal births. [file Image_1.TIFF]

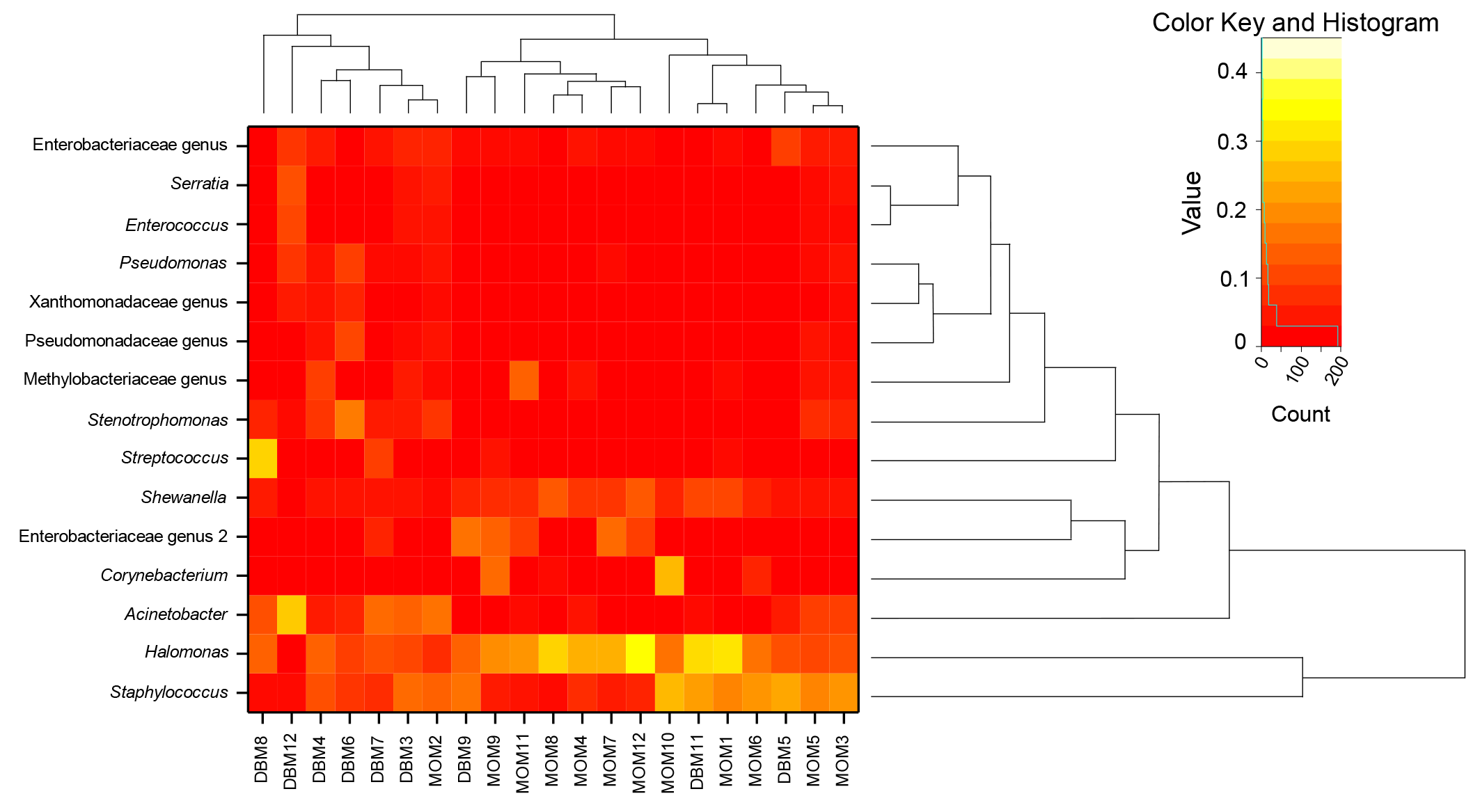

Supplement: FIGURE S2 — Heatmap showing the top 15 genera of each DBM sample, with the exception of replicates DBM 1, 2 and 11 (DBM 1 and 2 being a replicates of 3 and DBM 11 being a replicate of 12), and each MOM sample. [file Image_2.tif]
