# Supplementary material for: Personalization of the Microbiota of Donor Human Milk with Mother’s Own Milk
Source: Front Microbiol. 2017 Aug 3;8:1470. doi: 10.3389/fmicb.2017.01470 (PMC5541031; doi:10.3389/fmicb.2017.01470)
Supplement: Supplementary file 3 [file Table_1.DOCX]

**Supplementary Table 1.** Genera Comparison between C-Section and Vaginal Mode of Delivery.

| **Genera** | **C-Section mean** | **Vaginal mean** | **P-value** |
| --- | --- | --- | --- |
| *Acinetobacter* | 8.79E-02 | 1.25E-02 | 0.1235 |
| *Aggregatibacter* | -9.95E-05 | 6.34E-04 | 0.1158 |
| *Agrobacterium* | 2.58E-03 | 2.53E-04 | 0.05699 |
| *Candidatus Portiera* | 5.82E-06 | 2.77E-05 | 0.1131 |
| *Citrobacter* | 5.16E-05 | 7.29E-06 | 0.0895 |
| *Clostridium* | 2.07E-04 | 1.05E-03 | 0.06939 |
| *Comamonas* | 1.59E-05 | 6.38E-05 | 0.09814 |
| *Dorea* | 1.98E-04 | 5.90E-04 | 0.06613 |
| *Enterococcus* | 1.63E-02 | 2.59E-03 | 0.09824 |
| *Erwinia* | 4.00E-05 | 0.00E+00 | 0.02732 |
| *Halomonas* | 1.75E-01 | 3.20E-01 | 0.02302 |
| *Klebsiella* | 7.55E-05 | 2.60E-06 | 0.08498 |
| *Lactobacillus* | 4.95E-03 | 2.39E-02 | 0.04249 |
| *Macrococcus* | 1.97E-04 | 3.26E-05 | 0.1398 |
| *Prevotella 2* | 4.40E-03 | 8.74E-03 | 0.04413 |
| *Pseudomonas* | 2.08E-02 | 1.14E-03 | 0.03135 |
| *Ruminococcus* | 7.15E-04 | 2.242E-03 | 0.04146 |
| Unclassified Xanthomonadaceae genus | 5.12E-03 | 3.72E-04 | 0.1242 |
| Unclassified Bacilli genus | 7.68E-05 | 2.59E-06 | 0.09944 |
| Unclassified Bradyrhizobiaceae genus 2 | 3.11E-05 | 1.10E-05 | 0.09577 |
| Unclassified Clostridiaceae genus | 3.56E-05 | 2.82E-04 | 0.03386 |
| Unclassified Enterococcaceae genus | 7.48E-05 | 2.17E-05 | 0.02289 |
| Unclassified Lachnospiraceae genus | 2.84E-03 | 6.90E-03 | 0.1333 |
| Unclassified Lactobacillales genus | 1.14E-03 | 1.04E-04 | 0.1242 |
| Unclassified Lactobacillales genus 2 | 2.24E-03 | 1.80E-04 | 0.116 |
| Unclassified Methylophilaceae genus | 2.59E-05 | 5.96E-06 | 0.0763 |
| Unclassified Pseudomonadaceae genus | 1.06E-02 | 7.68E-04 | 0.1014 |
| Unclassified PYR10d3 genus | 7.42E-05 | 9.87E-06 | 0.1227 |
| Unclassified Sphingomonadales genus | 1.04E-04 | 8.97E-06 | 0.1073 |

*_P_*_-values determined by a Welch’s t-test comparing C-section to Vaginal deliveries. A_ *_p_* _value < 0.13 was used as inclusion criteria._
